# Reviews of Books

**Published:** 1921-10

**Authors:** 


### REVIEWS OF BOOKS.

**THE PROSPECTIVE MOTHER.** By J. Morris Slemons, M.D. New edition. (Messrs. D. Appleton & Co. Price 9s. net.)

The expressed object of Dr. Slemons' clearly written book is to provide for women "who have no special knowledge of medicine" answers to the questions which occur to them in the course of pregnancy. How far potted medical information should be provided for the layman and laywoman is a fruitful theme for controversy. The age we live in has a sweet tooth for a synopsis; and there is perhaps a tendency to mistake for education a superficial smattering of "general knowledge" on every conceivable question. It is thought correct that our children should know "something" (however scrappy) about botany and bootmaking, engineering and eugenics and Egyptology. Educationalists in their ardour leave no subject untouched lest the world should think them unaware of its existence; a surface skimming is removed and confidently dispensed as the "gist" of the matter; and the schoolboy recipient parades his encyclopædic knowledge before his baffled but admiring parents. A poet who is now somewhat neglected once wrote warningly of the danger of a little knowledge; what was true three hundred years ago is far truer to-day, when human information has increased enormously in extent and intricacy. No subject has widened and deepened its scope more than that which is the domain of the physician, and none is more full of pitfalls for the casual reader.

In the field of personal hygiene there are admittedly certain general principles which have established themselves as a result of long research and observation, and which can, and ought to, form part of the education of every citizen. Modern civilisation is essentially an artificial product; we all live under artificial conditions; our instincts are no longer safe guides as are the instincts of animals living in a state of nature; and instinct has often to be replaced by instruction. But instruction in general principles is one thing, and instruction in technical details is another; and the provision of information of the latter sort may easily do harm when it is meant to do good. The guidance of the prospective mother on approved lines is an especially important matter; and it may be said at once that in Dr. Slemons' book the general principles to which her attention should be directed, and the way in which her daily life should be temporarily modified, are indicated in a manner which is at once scientifically accurate and interesting. The first edition has already attained to a wide measure of popularity in America, and the new edition has been rewritten with a view to bringing the book abreast of the most recent advances in knowledge. The chapters on "The Care of the Body," "General Hygienic Measures," and "Preparations for Confinement" are eminently practical and suggestive. It must be left to the individual physician, however, to judge whether some of the material, at least, that is included is really within the scope of what can with safety and real advantage be told to average women without medical training—even to women who belong to the more highly educated classes. In the glossary, for example, one finds definitions of Cæsarean operation, chorionic membrane, chromatin, colostrum. Doubt may be felt as to whether the pregnant woman, with her already over-sensitive imagination and tendency to introspection, is likely to benefit from reading about the ailments of pregnancy.

The true guide for the expectant mother is, and must always be, not a text-book but a physician. As Dr. Slemons himself remarks, "Prospective mothers must be taught that they should be under competent medical supervision throughout pregnancy." This is the central fact which one would like to see brought home in preference to the most excellent teaching on the structure of the placenta. Let us hope that one day the public mind will grasp the need for this supervision, and that the time will come when for every expectant mother adequate supervision will be available, whether in the private consulting-room or in the ante-natal clinic. Dr. Slemons' book is written throughout with directness and lucidity; and while, as has been said, it must be left to the individual doctor to decide about the desirability of handing it to his lay patients, it would be difficult to find a more suitable introduction to the subject to set before the midwife, the nurse, or the medical student.—D. M. M.

**CLINICAL EXAMINATION OF THE NERVOUS SYSTEM.** By Monrad-Krohn. (H. K. Lewis & Co. Pp. 135. Price 6s. net.)

Neurological diagnosis is beset with terrors due to partial knowledge. Easily-forgotten anatomical and physiological facts are of supreme importance, and, unless the investigator has in mind a regular scheme of examination, important facts are apt to be overlooked and others misinterpreted—with the result that what should become clear remains obscure.

This little book lays stress on the importance of being in possession of all the facts before jumping to conclusions, and contains a warning against the fascinating but dangerous habit of "lightning-diagnosis." The author tells the student and reminds the practitioner what signs to look for, how to look for them, and what they mean. He gives in some detail an orderly system of examination; the tests to be applied are clearly described and their significance is explained in simple language. Electrical examination, diplopia, and vestibular tests receive special attention. The investigation of aphasia and apraxia is clearly described, and the explanation of their production is in keeping with modern views. Stress is laid upon the interdependence of neurology and psychiatry, and the Binet-Simon tests receive a description which, though short, suffices to define the landmarks of normal and subnormal mental development in the child.

**Reviews of Books—(*cont.*).**

A mastery of the contents of this small book should go far to clarify the conceptions of the man who is called upon to deal with a case of nervous disorder, and an examination of the patient according to this scheme should put the observer in possession of all the essential facts upon which to base a correct diagnosis. An occasional error in spelling and an unusually frequent use of the pronoun " one " add an element of expectancy to the reader's interest, but do not detract from the value of the book.—C. E. S.

**THE HERETIC : A STUDY OF TEMPERAMENT.** By J. MILLS WHITHAM. (George Allen and Unwin, Ltd. Price 8s. 6d. net.)

THIS is not a Freudian novel, but a story with a medical theme. Dedicated to Mr. H. A. Barker, it describes the boyhood and later struggles of the born bone-setter, the man in whose fingers lies a genius for manipulation. The son of a scholarly recluse, Raymond Verne is brought up on the heart of Exmoor in a lonely house, and his chief amusement is to catch and boil rabbits in order that he may study their skeletons. Would we had more such boys! They exist, but in small numbers. Raymond tries his hand on a dislocated sheep's leg, then on a farm-hand, with marked success. On the death of his father he becomes apprenticed to a notorious bone-setter in London, whose disorderly habits are in pathetic contrast to his peculiar genius for manipulative surgery. Pratt, the bone-setter, refuses to qualify, and is the despair, if not the victim, of the orthodox practitioner. Among this class Raymond's uncle is a flourishing example. He entreats the lad, not too tactfully, to start on the road which leads to Harley Street. In vain! Raymond worships his master, Pratt, the bone-setter, and determines to carry on the war and the tradition of the unregistered practitioner. When Pratt dies Raymond succeeds to his diminishing practice, but by native genius, intense study, pluck, and good fortune, more than repeats his master's success. He relieves a prize-fighter, and becomes known. A lady of title is his next success. He exchanges Bloomsbury for Mayfair, and becomes the rage. The drama of the story exists, of course, in the struggles of the hero with orthodox medicine. Unfortunately, his chief opponent, the aforesaid uncle, is little more than a stage figure, stiff with convention, etiquette, and philistinism. The story would be much better than it is if more subtlety had been given to the characters. Neither the hero nor his opponent presents more than the surface of his respective point of view. It is not made sufficiently clear why Raymond refuses to qualify, nor why the medical uncle refuses to consider the admitted successes of the osteopath. Obviously there is more than appears on the surface in the attitude of either camp; and here Mr. Barker, the dedicatee, might have given the author hints which would not only have improved the story, but made at least one party more intelligible to the general public. Even so, the book is readable enough, and the arrival of a bone-setter among the heroes of the contemporary novel is a tribute not to be mistaken to the position which the irregular practitioner has won for himself. Because it sheds a ray of light upon public ignorance and misunderstanding, the book is welcome. The story carries one through, and our only regret is that the conflict described is not treated with greater knowledge and subtler understanding. Mr. Mills Whitham has shown the way. Perhaps he or some emulative writer will pursue it further in another novel.—O. B.

**HYGIENE FOR HEALTH VISITORS, SCHOOL NURSES, AND SOCIAL WORKERS.** By C. W. HUTT, M.A., M.D., D.P.H. Second edition. (London: Methuen & Co., Ltd., 1921. Pp. 382, 83 illustrations. Price 12s. 6d. net.)

HEALTH visitors now form an important section of the staff of most sanitary authorities, and if it is necessary for them to possess much tact, patience, and similar personal qualities to ensure success, it is no less essential that they should be well trained in their work, and that they should have a sound and comprehensive knowledge of hygiene. The people with whom the health visitor has frequently to deal are sometimes prejudiced and biased, and, if opportunity presents itself, they are only too ready to criticise any ignorance which may be shown. To assist women training for these posts in the Public Health Services Dr. C. W. Hutt, Medical Officer of Health for the Metropolitan Borough of Holborn, has written a useful book, which will be of great service in giving those who read it a thorough understanding in the necessary subjects.

The beginning of this volume deals with the essentials of physiology, and it cannot be too strongly impressed on students that a knowledge of this subject is necessary before they can comprehend even elementary medicine; succeeding chapters deal with the various subjects included in the general term " hygiene." It is gratifying to notice the emphasis laid on such questions as the necessity for sufficient and adequate means for the storage of food, and the thorough way in which personal hygiene and the clothing of children is dealt with, though in connection with the latter subject we would like to have seen the now fashionable method of unduly exposing the arms and legs of children more strongly condemned. The section on drainage seems to be treated in rather a detailed manner, and it would appear that the intricacies of such things as soil pipes, gulley traps, manholes, and ferret spring drain cleaners concern the sanitary inspector rather than those for whom this book is written. Perhaps the best chapters of the volume are those dealing with infant mortality, and the care of infants and children; the subject is treated very well and fully, and if the knowledge contained in this section is imparted to mothers, it will do much to reduce the " maternal ignorance, which is the grand cause " of the mortality of infants.

What pleases us especially is the Appendix B, for here is given a good account of facts concerning cancer, which should be widely known, with special reference to malignant disease of the uterus and breast. It would be most easy, when asked by a

**Reviews of Books** (*cont.*).

middle-aged woman complaining of irregular uterine hæmorrhage, for a health visitor to say that nothing was the matter, to maintain that such symptoms were connected with the menopause, and to prophesy "smooth things," because "the people love to have it so"; and we welcome the advice given by Sir Francis Champneys, Chairman of the Central Midwives Board, and quoted by the author: "All women (such as nurses and midwives, but not only they), who are especially liable to be consulted on these matters, should avoid expressing any opinion of their own, but should advise the inquirer to go at once to a properly qualified medical practitioner, and insist on being examined." It would be well in future editions of the book to give this appendix the dignity of a chapter and the accompanying large print, for it is only by such methods of propaganda that we can hope to reduce the ominous increase of malignant disease.

The book as a whole is well written, and by the judicious avoidance, as far as possible, of technical terms, should be easily intelligible to those for whom it is intended; that it is thoroughly up-to-date is evidenced by such sections as those dealing with encephalitis lethargica, vitamins, and the grading of milk.—J. V. A. S.

